# Selection of validated hypervariable regions is crucial in 16S-based microbiota studies of the female genital tract

**DOI:** 10.1038/s41598-018-27757-8

**Published:** 2018-06-26

**Authors:** Simon Graspeuntner, Nathalie Loeper, Sven Künzel, John F. Baines, Jan Rupp

**Affiliations:** 1 Department of Infectious Diseases and Microbiology, University of Luebeck, 23538 Luebeck, Germany; 2 Max Planck Institute for Evolutionary Biology, 24306 Ploen, Germany; 3 Institute for Experimental Medicine, Christian-Albrechts-University of Kiel, 24105 Kiel, Germany; 4 German Center for Infection Research (DZIF), partner site Hamburg-Luebeck-Borstel, Germany

## Abstract

Next-generation sequencing-based methods are extensively applied in studies of the human microbiota using partial 16 S rRNA gene amplicons. However, they carry drawbacks that are critical to consider when interpreting results, including differences in outcome based on the hypervariable region(s) used. Here, we show that primers spanning the V3/V4 region identify a greater number of taxa in the vaginal microbiota than those spanning the V1/V2 region. In particular, taxa such as *Gardnerella vaginalis*, *Bifidobacterium bifidum* and *Chlamydia trachomatis*, all species that influence vaginal health and disease, are not represented in V1/V2-based community profiles. Accordingly, missing or underestimating the frequency of these species overestimates the abundance of other taxa and fails to correctly assess the bacterial diversity in the urogenital tract. We elaborate that covering these taxa using the V3/V4 region leads to profound changes in the assignment of community state types. Altogether, we show that the choice of primers used for studying the vaginal microbiota has deep implications on the biological evaluation of the results.

## Introduction

Human cavities are colonized with microorganisms and these form in their entity the human microbiome, which became intensively studied when next-generation sequencing (NGS) emerged. NGS offered the opportunity of cultivation-independent assessment of microbial communities and therefore revealed a multitude of thus far unknown bacteria. Since the first report on the analysis of the vaginal microbiota employing NGS^1^, amplification of partial sequences of the bacterial 16 S gene with primers spanning hypervariable regions is the common method to describe vaginal bacterial populations. In recent years, research has shown that the vagina of humans can be colonized by distinct bacterial communities, often dominated by a *Lactobacillus* species^1^. However, also diverse communities are observed^1^ and some communities display a significant fraction of *Gardnerella vaginalis*^2^. These different compositional states of bacterial communities were termed community state types (CSTs) by Gajer *et al*. subsequently^3^. Importantly, a rising number of reports show that the vaginal microbiota play an important role in the acquisition and prevention of sexually transmitted diseases and their long term sequels^2,4–8^. However, as it frequently appears, the amplified region used varies from study to study. While many studies use the primers 27 F and 338 R spanning the V1/V2 hypervariable region (e.g.^1,3^), others choose the V3/V4 region (e.g.^2,9^). It has been shown that the choice of the primers used for amplification can introduce bias to the results achieved when applying 16S amplicons for microbiome studies^10–12^. Thus, it is important to consider the validity of the primers for given sample type being analysed^13^. Despite a report of the V1/V2 region being inappropriate for targeting vaginal bacteria such as *Gardnerella vaginalis*^14^, no study to date compared the relative performance of V1/V2 to another hypervariable region for vaginal samples. To address this uncertainty, we studied a set of cervical swabs derived from different clinical sites and compared the results when using the V1/V2 spanning primers (27 F/338 R) to the same samples amplified using primers spanning the V3/V4 (319 F/806 R) hypervariable region. This analysis contributes to the standardisation of vaginal microbiota studies and provides critical insight into the comparability between studies.

## Results

### The V3/V4 region identifies more taxa and displays higher diversity than the V1/V2 region

**Figure 1:**
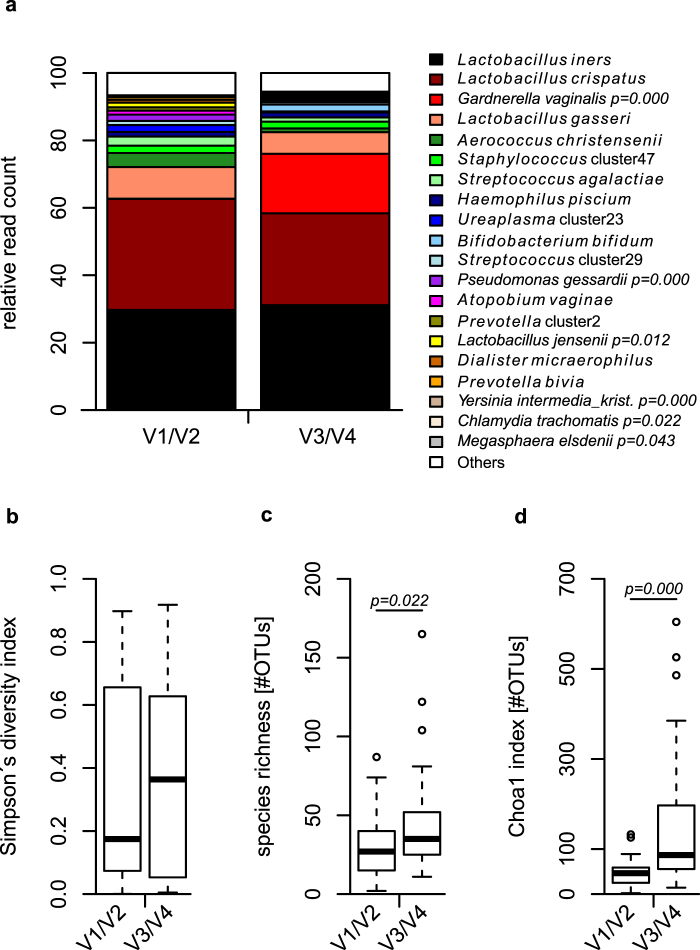
Relative read count of several bacterial taxa and bacterial alpha diversity differ according to the region used. The V3/V4 region shows significantly enhanced relative read count of taxa such as *Gardnerella vaginalis* and *Chlamydia trachomatis* in comparison to the V1/V2 region. Inversely, *Lactobacillus jensenii*, *Pseudomonas gessardii* and *Megasphaera elsdenii* are significantly reduced in their relative read count in the V3/V4 data set (**a**). Observed and estimated total number of OTUs within the samples are significantly higher when comparing the V3/V4 region with the V1/V2 data set using operational taxonomic units clustered with a 97% global identity threshold (**b**–**d**). Statistical comparisons were performed using Wilcoxon rank-sum test.

In total we sequenced the V1/V2 and V3/V4 regions for 38 samples, with a normalized sequencing coverage of 5000 reads per sample after data processing. Sequence classification was performed using the same approach^15^ for both data sets and we subsequently compared the resulting taxa identified. Among the most abundant taxa, we observed *Gardnerella vaginalis*, and *Chlamydia trachomatis* to be significantly enhanced in the V3/V4 data set compared to the V1/V2 region, while other taxa, such as *Lactobacillus jensenii*, *Pseudomonas gessardii*, and *Megasphaera elsdenii* are correspondingly decreased (Fig. 1a and Supplementary Table S1).

We further applied several methods for estimation of alpha diversity using OTUs clustered with a similarity threshold of 97%. While Simpson’s diversity index reveals a non-significant trend to be enhanced (Fig. 1b), observed (Fig. 1c) and estimated total (Fig. 1d) number of OTUs show significant higher values for the V3/V4 in comparison to the V1/V2 region.

### The V3/V4 region identifies community state types with characteristic species lacking in the V1/V2 region

**Figure 2:**
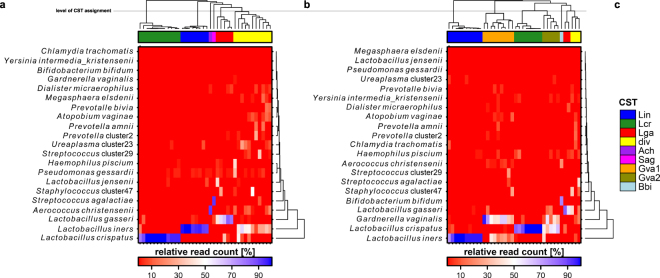
Heatmaps based on relative read counts of the major bacterial taxa including clustering of the urogenital microbial communities into several CSTs above the heatmaps. While the samples cluster into 6 CSTs within the V1/V2 data set (**a**), the V3/V4 region displays partially different CSTs with reduction of the proportion or elimination of the CSTs present in the V1/V2 region (**b**). The color coding for the CSTs is given in (**c**). The clusters are named based on characteristic taxa: Lin: *Lactobacillus iners*, Lcr: *L*. *crispatus*, Lga: *L*. *gasseri*, div: diverse, Ach: *Aerococcus christensenii*, Sag: *Staphylococcus agalactiae*, Gva: *Gardnerella vaginalis*, Bbi: *Bifidobacterium* *bifidum*.

**Figure 3:**
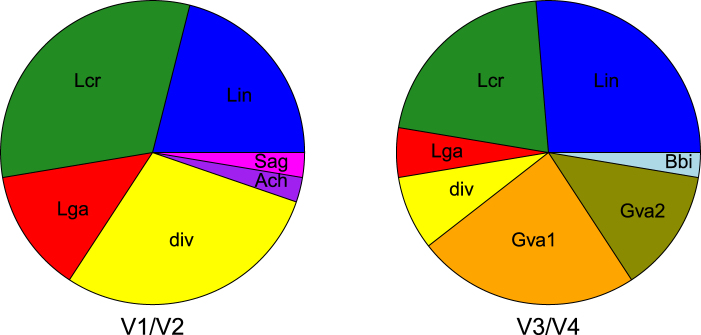
Proportion and presence of assigned CSTs change depending on the 16S region used for amplification. The two regions reveal different CSTs. The proportion of *Lactobacillus*-dominated and diverse CST appears lower while CSTs characterized by the presence of *Gardnerella vaginalis* are prominent when using the V3/V4 region in comparison to the V1/V2 data set (Pearson’s chi square test: p = 0.002). The CSTs are named according to characteristic species of each cluster: Lin: *Lactobacillus iners*, Lcr: *L*. *crispatus*, Lga: *L*. *gasseri*, div: diverse, Ach: *Aerococcus christensenii*, Sag: *Staphylococcus agalactiae*, Gva: *Gardnerella vaginalis*, Bbi: *Bifidobacterium* *bifidum*.

We observe a total of six community state types (CSTs) present in the data set using the V1/V2 region. Five of them are characterized by a dominant species, which are either *Lactobacillus iners* (Lin CST), *L*. *crispatus* (Lcr CST), *L*. *gasseri* (Lga CST), *Aerococcus christensenii* (Ach CST) or *Streptococcus agalactiae* (Sag CST), respectively. One CST is more diverse (div CST) (Fig. 2a) and not dominated by a single species. In contrast, the number of CSTs dominated by *L*. *crispatus* and *L*. *gasseri* appears lower based on the V3/V4 region (Fig. 3). While the div CST and the CSTs dominated by *A*. *christensenii* and *S*. *agalactiae* are either less frequent or not present in the V3/V4 data set, two CSTs with characteristic *G*. *vaginalis* abundance (Gva1 and Gva2 CST) and one CST dominated by *Bifidobacterium bifidum* (Bbi CST) are appearing (Fig. 2b). The differences between the two regions appear to be significant (Fig. 3, p < 0.01, chi-square test).

**Figure 4:**
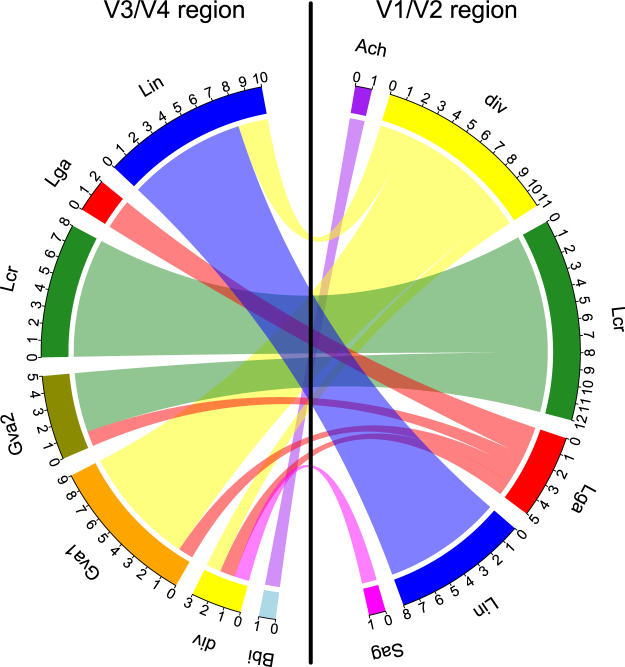
A considerable amount of samples display a CST when the V1/V2 region is used that appears as a different CST when using the V3/V4 region. The migration plot shows the number of samples belonging to each CST within both data sets and displays the connections between samples with different CSTs between the regions. Thus, the plot shows e.g. that a certain number of samples the V1/V2 region displays as Lcr, Lga and div CST are shown to belong to the Gva1 and Gva2 CST when using the V3/V4 region. The CSTs are named according to characteristic species of each cluster: Lin: *Lactobacillus iners*, Lcr: *L*. *crispatus*, Lga: *L*. *gasseri*, Gva: *Gardnerella vaginalis*, div: diverse, Bbi: *Bifidobacterium* *bifidum*, Ach: *Aerococcus christensenii*, Sag: *Staphylococcus agalactiae*.

We next analysed to which CST a sample belongs according to the different regions. Following the constructed migration plot (Fig. 4), we observe that parts of the samples belonging to the Lga CST in the V1/V2 region cluster to the Gva1/2 and div CST using the V3/V4 data set, while for the Lcr CST (V1/V2) we observe a transition to the Gva2 CST (V3/V4). The diverse CST from the V1/V2 data set mostly switches to the Gva1 CST in the V3/V4 region. The samples assigned to the singletons Ach CST and Sag CST from the V1/V2 data set appear in different CSTs using the V3/V4 region.

We additionally performed qPCR detection of *G*. *vaginalis* in a subset of samples. Thus, we could prove the presence of *G*. *vaginalis* in all samples with a Gva CST. We further tested two samples which were negative for *G*. *vaginalis* reads in the sequencing and they were negative in the qPCR as well (Supplementary Fig. S1).

### Only the V3/V4 region identifies the major sexually transmitted pathogen *Chlamydia trachomatis*

*C*. *trachomatis* is the most prevalent sexually transmitted disease worldwide and the major reason for severe pathological consequences such as pelvic inflammatory disease. We specifically analysed read numbers classified as *C*. *trachomatis*. While we observe no *C*. *trachomatis* reads using the V1/V2 region, *C*. *trachomatis* is clearly identified using the V3/V4 region (Fig. 1 and Supplementary Table S1), with four samples displaying *C*. *trachomatis* read numbers ranging from 22 to 526 (0.44% to 10.52% relative read count) (Table 1). We additionally confirmed the presence of *C*. *trachomatis* in these samples using conventional qPCR (Table 1). Three of the *C*. *trachomatis* positive samples appear with a change in their CST between the two regions (Supplementary Table S2). However, both regions identify certain bacterial taxa associated with *C*. *trachomatis* infection in an indicator species analysis using infection status as grouping variable (Supplementary Tables S3 and S4).

**Table 1:** Samples with reads classified to belong to *Chlamydia trachomatis* using the V1/V2 and the V3/V4 region.

Sample	Reads V1/V2 (relative read count [%])	Reads V3/V4 (relative read count [%])	LightMix® *C*. *trachomatis*
B044	0 (0.00)	503 (10.06)	positive
B048	0 (0.00)	526 (10.52)	positive
B074	0 (0.00)	24 (0.48)	positive
B087	0 (0.00)	22 (0.44)	positive
Total	0 (0.00)	1075 (0.57)	NA

## Discussion

Numerous combinations of primer pairs targeting different regions of the bacterial 16S gene have been employed to identify the composition of bacterial communities by NGS. Previous research shows that different regions yield contrasting results when applied on the same samples^10,12,16^. However, prior to this study the representativeness of commonly used 16S rRNA gene primers pairs was not assessed for the lower urogenital tract.

It is important to note that in general partial 16S sequences are critical when it comes to classification as different taxa can be classified to different levels. While we are not able to classify all taxa to species level, this is possible for many species within the urogenital tract, enabling us to discuss the detailed taxonomic analysis we provide here.

Our study indicates that the V1/V2 region fails to identify several important species inhabiting the lower urogenital tract (e.g. *B*. *bifidum*, *G*. *vaginalis* and *C*. *trachomatis*), which is in line with a previous report^14^. Thus, the usage of the 27 F/338 R primer pair fails to detect bacteria important to vaginal health and disease. In contrast, the primers commonly used for amplifying the V3/V4 hypervariable region do not have this limitation^17^.

The most drastic changes we observe between the two regions are due to the abundance of *G*. *vaginalis*. The V3/V4 region determines this species as an characteristic component of many bacterial communities in this data set, which is additionally confirmed by qPCR quantification. In contrast, the V1/V2 region lacks the *G*. *vaginalis* detection, which may lead many samples to deviate from their true underlying community structure. This is noticeably shown by a considerable amount of samples having a Lcr or Lga CST based on V1/V2 which are clustering into a CST with high relative read counts of *G*. *vaginalis* (Gva1 and Gva2 CST) based on V3/V4. These differences in CST assignment are important to consider, as the biological function in defense to pathogens largely depends on microbial composition. Within the commensal vaginal microbiome, lactobacilli are a major maintainer of vaginal balance and health, as they are capable of producing lactic acid and therefore lowering the pH^18^. Indeed, it was recently shown that a CST with high relative amounts of *L*. *crispatus* is associated with a decreased risk of *C*. *trachomatis* transmission from infected partners^6^ and that *L*. *crispatus* shows inhibitory capacity on the infectivity of *C*. *trachomatis in vitro*^5^. Further, the ability of the bacterial community to trap HIV is dependent on the CST present in the vagina^2^ and a protective role of *L*. *crispatus*-dominated CST against *Escherichia coli* in the vagina was shown^19^. The interpretation of such studies is largely driven by the primers used, as the number of *Lactobacillus*-dominated samples seems to be overestimated with the V1/V2 region as our data show. Thus, a recent study by Callahan *et al*. focusing on the presence of lactobacilli vs. *Gardnerella* in the preterm birth microbiota^20^, would have yielded severely erroneous results had they used the V1/V2 region primers. The missing *C*. *trachomatis* reads by using the V1/V2 primers further emphasizes the importance of using primers for V3/V4 or another alternative for urogenital microbiota studies.

However, while the V1/V2 region appears to be characterized by under-representing vaginal pathogens such as *G*. *vaginalis* and *C*. *trachomatis* on the one hand, it is also seems to present other important vaginal pathogens more prominent as they are in reality. As of example, *Ureaplasma urealyticum* is an often detected pathogen in the vagina^21^ and seems to be over-estimated using the V1/V2 region. In combination, the skewed representation of important vaginal bacteria might shift the focus of pathogens in a misleading direction in microbiota analysis determined by the V1/V2 region.

Considering the above-mentioned importance of CST composition in the defense against infectious diseases, a particular focus needs to be set on a complete and comparable assignment of CSTs. While Robinson *et al*. guide researchers towards standardization of their protocols in microbiota studies using 16S genes in general, and point out the importance of the primer choice^13^, we here show that the V3/V4 region seems to perform much better than the V1/V2 region for urogenital studies in females when using the common primer pairs as outlined in the method section. Of note, special formulations of primers have also been developed to improve mismatch-based inadequacy^14^ and have been used for microbiota studies^22,23^. However, this coincides with reduced efficiency and specificity during amplification^14^, and the validity of such mixtures has been called into question^24^. With our study we therefore aim to contribute to the awareness of accurate study design in microbiota studies of the urogenital tract of females.

## Methods

### Sample collection

Cervical swabs from healthy females were collected at different clinical sites in Germany, avoiding contamination by other parts of the vagina or the body. All study participants were informed about the purpose of the study and usage of the data and signed a declaration of consent for participation. The study was approved by the ethics committee of the University of Lübeck with the reference number 11–185. All methods were performed in accordance with the relevant guidelines and regulations. Swabs were transported on dry ice and stored in Universal Transport Medium (UTM, COPAN Diagnostics) at −80 °C.

### DNA-isolation

One swab per woman was vortexed at highest speed for 1 min. DNA was isolated from 1 ml of the remaining buffer using the MoBio PowerSoil^®^ Kit following the instructions from the manufacturer’s protocol. We introduced a 2 h incubation with OB-Protease at 50 °C followed by homogenisation of the sample using a MoBio PowerLyzer^®^, both prior to the first centrifugation step. Furthermore, double amounts of solutions C2, C3, and C4 were applied due to the high sample volume. Isolated DNA was stored at −20 °C.

### Sequencing

We amplified two partial 16S gene sequences from each isolated DNA in separate PCR-reactions. We used the primer pair 27 F/338 R (27 F: 5′-AGAGTTTGATCCTGGCTCAG-3′/ 338 R: 5′-TGCTGCCTCCCGTAGGAGT-3′) to amplify the V1/V2 region, and the primer pair V3F/V4R (V3F: 5′-CCTACGGGAGGCAGCAG-3′/ V4R: 5′-GGACTACHVGGGTWTCTAAT-3′) for the V3/V4 hypervariable region from the same sample. Sequencing was performed on a MiSeq sequencer (Illumina). All primers contained unique identifier sequences (barcodes) to distinguish between the samples following published methods^25^ with an optimized primer design^26^.

### Data processing

The raw data were processed using mothur^27^ version 1.38.1 following a script we optimized based on our previous studies^28^. In brief, we removed all sequences with deviations from the original primer and barcode sequences. We removed all sequences with ambiguous bases, a length greater than the amplified fragment, and a homopolymer length greater than 12. The sequences were aligned with the SILVA references database^29^, chimera were removed using the uchime algorithm^30^. We clustered the processed sequences into operational taxonomic units (OTU) with a global identity threshold of 97% or performed classification based analysis. Species level classification was performed using STIRRUPS using either the V1/V2 or the V3 region^15^.

### Statistical analysis

All statistical analysis and graphical visualisations were carried out in R version 3.2.2^31^. Differences in relative read count of taxa and alpha diversity were tested using Wilcoxon rank sum test. Simpson’s diversity index, species richness, and chao1 index were used as a measure for alpha diversity and computed using R package vegan^32^ based on OTUs. Heatmaps were constructed with R package BoutrosLab.plotting.general^33^ using the most abundant taxa. Community state types (CSTs) were assigned by euclidean distances based on relative read counts of taxa with complete linkage clustering and CSTs were assigned on the same level of the hierarchical clustering to enable unbiased CST building. Differences in the proportion of CSTs between the two methods were tested using chi-square testing. A migration plot showing the CST changes between the regions was elaborated using R packages migest^34^ and circlize^35^.

### Diagnosis of Chlamydia trachomatis

Samples with reads classified as *C*. *trachomatis* in the microbiota sequencing were additionally tested by a specific PCR test to prove infection with *C*. *trachomatis* (LightMix^®^ Kit *C*. *trachomatis*, TIB MOLBIOL) following the manufacturer’s instructions.

### Identification of bacterial taxa associated with *C*. *trachomatis*-positive samples

We performed an indicator species analysis using the R package indicspecies^36^ for each region separately to identify bacterial taxa which associate with *C*. *trachomatis* infection with infection status as grouping variable.

### Quantification of Gardnerella vaginalis

We quantified the abundance of *G*. *vaginalis* using qPCR by calculating number of bacteria within a sample using a standard curve generated from a serial dilution with known colony forming units corresponding to DNA-content. The primer sequences used were taken from elsewhere^37^ (forward primer sequence: CGCATCTGCTAAGGATGTTG; reverse primer sequence: CAGCAATCTTTTCGCCAACT).

### Data availability

The datasets analyzed during the current study are available at the European Nucleotide Archive (ENA) with the accession number PRJEB23508.

## Electronic supplementary material

Supplementary Data
