# Supplementary material for: Selection of validated hypervariable regions is crucial in 16S-based microbiota studies of the female genital tract
Source: Sci Rep. 2018 Jun 26;8:9678. doi: 10.1038/s41598-018-27757-8 (PMC6018735; doi:10.1038/s41598-018-27757-8)
Supplement: Supplementary file 1 — Supplementary Data [file 41598_2018_27757_MOESM1_ESM.pdf]

Selection of validated hypervariable regions is crucial in 16S-based microbiota studies of the female genital tract

Simon Graspeuntner[a], Nathalie Loeper[a], Sven Künzel[b], John F. Baines[b,c], Jan Rupp[a,d*]

[a]Department of Infectious Diseases and Microbiology, University of Luebeck, 23538 Luebeck, Germany
[b]Max Planck Institute for Evolutionary Biology, 24306 Ploen, Germany
[c]Institute for Experimental Medicine, Christian-Albrechts-University of Kiel, 24105 Kiel, Germany
[d]German Center for Infection Research (DZIF), partner site Hamburg-Luebeck-Borstel, Germany

Running title: 16S region in female genital tract microbiota studies

*Corresponding author:
Jan Rupp, MD
Department of Infectious Diseases and Microbiology
University Hospital Schleswig-Holstein/ Campus Luebeck
University of Luebeck
Ratzeburger Allee 160
23538 Luebeck, Germany
Tel.: +49 (0)451/ 500- 45300
Fax.: +49 (0)451/ 500- 45304
e-mail: Jan.Rupp@uksh.de

Supplementary Table S1: Relative read count according to Figure 1

Taxon	V1V2	V3V4	p-value
Lactobacillus iners	29.724	31.115	0.726
Lactobacillus crispatus	32.929	27.256	0.390
Gardnerella vaginalis	0.054	17.646	0.000
Lactobacillus gasseri	9.386	6.432	0.413
Aerococcus christensenii	4.171	1.109	0.789
***Staphylococcus* cluster47**	2.177	2.003	0.855
Streptococcus agalactiae	2.706	1.243	0.483
Haemophilus piscium	1.374	1.487	0.923
***Ureaplasma* cluster23**	2.017	0.303	0.199
Bifidobacterium bifidum	0.013	2.032	0.308
***Streptococcus* cluster29**	1.238	0.707	0.174
Pseudomonas gessardii	1.862	0.020	0.000
Atopobium vaginae	1.169	0.633	0.469
***Prevotella* cluster2**	0.992	0.495	0.624
Lactobacillus jensenii	1.386	0.001	0.012
Dialister micraerophilus	0.839	0.486	0.462
Prevotella bivia	0.782	0.312	0.820
Yersinia intermedia_kristensenii	0.000	0.616	0.000
Chlamydia trachomatis	0.000	0.567	0.022
Megasphaera elsdenii	0.545	0.001	0.043
**Others**	6.637	5.539	NA

Supplementary Table S2: Community state types of C. trachomatis positive samples according to the different regions

Sample	CST V1/V2	CST V3/V4
**B048**	div	Lin
**B044**	div	div
**B074**	div	Gva1
**B087**	div	Gva1

Supplementary Table S3: Indicator species analysis[#] for *C. trachomatis* positive samples using the V1/V2 region

V1/V2	A*	B**	p-value
Streptococcus(100);cluster29(100);	0.97	0.75	0.02
Staphylococcus(100);cluster47(100);	0.93	0.75	0.03
Veillonella(100);montpellierensis(100);	0.83	0.75	0.01
Megasphaera(100);elsdenii(100);	0.71	0.75	0.005
Granulicatella(100);elegans(100);	1.00	0.5	0.01
Haemophilus(100);parainfluenzae(100);	0.99	0.5	0.01
Lachnospiraceae(100);BVAB1(100);	0.99	0.5	0.015
Fusobacterium(100);cluster48(100);	0.98	0.5	0.02
Prevotella(100);amnii(100);	0.89	0.5	0.03
Sneathia(100);sanguinegens(100);	0.84	0.5	0.03
Eggerthella(100);sinensis(100);	0.78	0.5	0.04
Ureaplasma(100);cluster23(100);	0.8255	0.75	0.025
Atopobium(100);vaginae(100);	0.8053	0.5	0.045

[#]Indicator species analysis shows the taxa which are significantly associated with infection status (i.e. associated with samples that were tested positive for C. trachomatis as outlined in Table 1).

*: ratio of total number reads of the respective taxon appearing in the *C. trachomatis* positive samples.

**: ratio of samples displaying reads of the respective taxon.

Supplementary Table S4: Indicator species analysis[#] for *C. trachomatis* positive samples using the V3/V4 region

V3/V4	A*	B**	p-value
Chlamydia(100);trachomatis(100);	1.00	1	0.005
Streptococcus(100);cluster29(100);	0.99	0.75	0.005
Veillonella(100);montpellierensis(100);	0.98	0.75	0.005
Sutterella(100);sanguinis(100);	0.93	0.75	0.01
Atopobium(100);vaginae(100);	0.93	0.75	0.02
Ureaplasma(100);cluster23(100);	0.88	0.75	0.035
Dialister(100);propionicifaciens(100);	0.70	0.75	0.03
Granulicatella(100);elegans(100);	1.00	0.5	0.005
Lachnospiraceae(100);BVAB1(100);	0.98	0.5	0.03
Haemophilus(100);parainfluenzae(100);	0.95	0.5	0.015
Prevotella(100);amnii(100);	0.91	0.5	0.045
Mycoplasma(100);hominis(100);	0.86	0.5	0.035
Delftia(100);acidovorans_lacustris_tsuruhatensis(100);	0.69	0.5	0.05
Clostridiales(100);BVAB2(100);	0.53	0.5	0.05

[#]Indicator species analysis shows the taxa which are significantly associated with infection status (i.e. associated with samples that were tested positive for C. trachomatis as outlined in Table 1).

*: ratio of total number reads of the respective taxon appearing in the *C. trachomatis* positive samples.

**: ratio of samples displaying reads of the respective taxon.

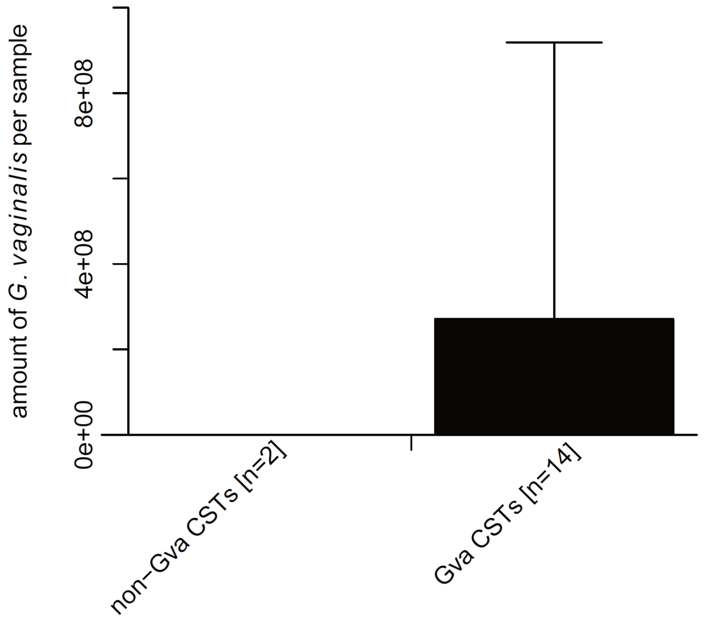

Supplementary Figure S1: The Gva CSTs contain a considerable amount of *G. vaginalis.* We performed qPCR detection of G. vaginalis in a subset of samples. Thus, we could prove the presence of *G. vaginalis* in all samples with a Gva CST. We further tested two samples which were negative for *G. vaginalis* reads in the sequencing and they were negative in the qPCR as well.
